# A Self-Propelled Traveling-Wave Linear Ultrasonic Motor Based on End Excitation

**DOI:** 10.3390/mi17040418

**Published:** 2026-03-29

**Authors:** Danhong Lu, Wenjian Qian, Nan Sun, Yao Chen, Xiaoxiao Dong, Bowen Chang

**Affiliations:** 1 School of Electric Power Engineering, Nanjing Institute of Technology, Nanjing 211167, China; y00450230728@njit.edu.cn (W.Q.); y00450230633@njit.edu.cn (N.S.); y00450230509@njit.edu.cn (Y.C.); 2 School of Electrical and Power Engineering, Hohai University, Nanjing 210024, China; dongxiaoxiao@hhu.edu.cn; 3 Engineering Training Center, School of Applied Technology, Nanjing Institute of Technology, Nanjing 211167, China; x00234220205@njit.edu.cn

**Keywords:** linear ultrasonic motor, traveling wave, piezoelectric ceramic, standing wave ratio, damping material

## Abstract

Ultrasonic motors have attracted considerable attention in precision actuation applications because of their advantages over conventional electromagnetic motors, such as compact structure, high positioning accuracy, immunity to electromagnetic interference, noise-free operation, and suitability for low-temperature environments. However, conventional traveling-wave linear ultrasonic motors usually rely on boundary constraints to establish stable traveling waves, which may limit their structural flexibility and self-propelled capability. To address this issue, this paper proposes a free-boundary traveling-wave linear ultrasonic motor capable of realizing self-propelled motion. The motor features a projection structure at each end of the stator. Two piezoelectric ceramics are placed at one end for excitation, while a damping material is arranged at the other end for energy absorption. This design enables the motor to generate traveling waves without requiring fixed boundary conditions. The motor operates in the B(3,1) out-of-plane vibration mode to enhance the energy absorption capacity of the non-excited end and reduce its standing wave ratio (SWR). A finite element model of the motor is established to investigate its vibration characteristics. In addition, a novel method for estimating the standing wave ratio is proposed by using piezoelectric ceramics attached to the motor surface, replacing the traditional calculation approach. A prototype is fabricated to verify the feasibility of the proposed design. Experimental results show that the prototype achieves a minimum SWR of 1.81, a no-load speed of 42.1 mm/s, and a maximum output force of 0.465 N. These results confirm the feasibility of the proposed scheme and provide a new approach for the design of free-boundary traveling-wave linear ultrasonic motors.

## 1. Introduction

Ultrasonic motors cause microscopic deformation of the piezoelectric material through the inverse piezoelectric effect of piezoelectric ceramics and ultrasonic vibration of stators through electromechanical coupling [1,2,3,4,5]. As a novel type of microelectromechanical system, ultrasonic motors possess several advantages over traditional electromagnetic motors, including high precision, low noise emission, flexible design methods, immunity to electromagnetic interference, noise-free driving, and the ability to be built in cryogenic environments, etc. [6,7,8,9,10,11]. The advantages above enable ultrasonic motors to be widely used in various working environments, such as aerospace [12,13], surgical procedures [14,15], miniature robotics [16,17], optical systems [18] and precision instruments [19].

In contrast to standing-wave ultrasonic motors, which move obliquely linearly [20], each particle in a traveling-wave-type ultrasonic motor displays continuously changing vibration phases. This results in elliptical motion trajectories that drive loads as simple harmonic motion wave peaks spread over space and impart energy to the particles [21]. Designing and manufacturing linear ultrasonic motors based on traveling waves is challenging due to the blockage and reflection of traveling waves at the end boundary when transmitted in non-enclosed structures [22]. Numerous experts have developed various linear traveling-wave ultrasonic motors to address this issue.

Liu et al. [23] designed a stator with a ring beam structure to reduce the reflection of traveling waves. Exciting two sets of piezoelectric ceramics can synthesize traveling waves to generate two sets of standing waves with a phase difference of 90° within the stator. Differences in the resonance frequencies of the two sets of standing waves can affect the generation of traveling wave components, preventing a proper elliptical trajectory on the driving surface. The prototype achieved a maximum speed of only 15 mm/s, indicating that the output performance was still limited. This approach requires strict structural symmetry of the stator. 

Some motors utilize dual piezoelectric transducers to generate traveling waves in stators, but the output thrust of the motors is generally low [24], with motors reporting a maximum output thrust of only 0.4 N. Kondo et al. [25] proposed a miniature traveling-wave ultrasonic linear motor using bimorph transducers, but the prototype achieved only 0.4 N maximum thrust and 154 mm/s maximum speed, with an efficiency of approximately 0.3%, a slip of 0.7, and an SWR of about two. These results indicate that, although the motor realized miniaturization, its output force, energy efficiency, and traveling-wave purity still remained limited. Z. Wen et al. [26] introduced bimorph transducers with a groove-tooth beam structure, which generates traveling waves using a phase difference driving method, facilitating the miniaturization of the stator. Although the standing wave ratio (SWR) was reduced to about 1.5 through phase adjustment, the stator still exhibited predominantly standing wave characteristics at both ends, while the traveling wave was mainly concentrated in the central narrow beam region. This indicates that the traveling-wave purity of the motor was still limited.

Fixed damping materials are placed at the ends of stators to absorb reflected traveling waves in some motors. Tominaga et al. [27] developed a linear ultrasonic motor with a ridge-shaped cross-section using high-damping materials and rectangular waveguides, but its output performance remained limited, with a maximum thrust of only 0.15–0.17 N. Moreover, the experimental prototypes were still relatively large, including a 1 m-long PMMA waveguide and a 29 × 54 × 149 mm aluminum structure. In addition, the standing wave ratio was highly sensitive to the terminal absorbing condition, varying from 18 to 1.2 with different terminating materials, which indicates that stable generation of a high-quality traveling wave was not easy to achieve. Y. Ting et al. [28] designed a short-beam linear piezoelectric motor with wedge-shaped reduction mechanisms at both ends with a large cross-sectional area, effectively reducing the reflection of traveling waves. Sufficient plastic deformation must occur within the damping material to absorb energy effectively. The measured traveling-wave peak amplitude was only about 0.38 μm at 300 Vpp, indicating that the vibration amplitude of the motor remained relatively limited. Due to stators’ unique excitation and energy absorption mechanisms, generating traveling waves in these materials often requires external boundary constraints. This approach not only compromises the flexibility of the motor but also limits its potential applications.

This paper proposes a linear ultrasonic motor of the traveling wave type using a thin-plate-type stator featuring protruding structures arranged at both ends. Two rectangular piezoelectric ceramics are placed on one side of the stator, operating in torsional vibration mode. An AC voltage with a phase difference of 180° is applied to excite two rows of incident traveling waves with a phase difference of 180°. A damping material is placed on the other side of the stator to absorb the energy of the incident traveling waves and reduce the reflected traveling waves. For the traveling-wave linear motor to drive the rotor and become self-propelled, the motor must operate under free boundary conditions at both ends while maintaining a compact structure. The excitation of vibrations and absorption of reflected traveling waves cannot be contingent upon external constraints. The vibration modes of stators greatly influence the energy absorption effect of damping materials. This paper utilizes the B31 mode of the stator to induce shear deformation within the damping material perpendicular to the direction of wave propagation. Experiments show that the damping material can achieve sufficient relative displacement to significantly reduce the stator’s standing wave ratio in this operational mode.

## 2. Structure of the Motor

### 2.1. Structure of Proposed Ultrasonic Motor

**Figure 1:**
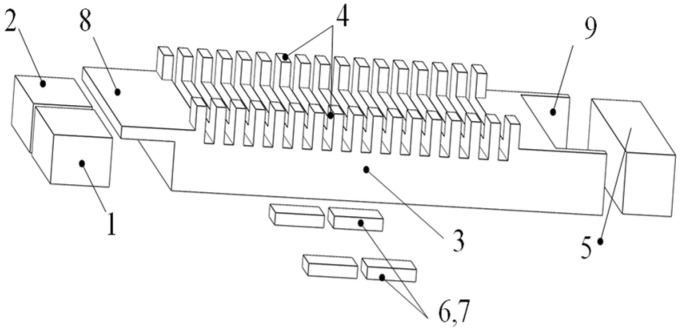
Structure of the ultrasonic motor: 1, 2—piezoelectric ceramics for excitation; 3—stator; 4—driving feet; 5—damping material; 6, 7—detection piezoelectric ceramics; 8—left protruding structure; 9—right protruding structure.

As depicted in Figure 1, this is an anatomical view of the ultrasonic motor, which comprises two excitation piezoelectric ceramics, two detection piezoelectric ceramics, a stator, two rows of driving teeth, and passive damping.

### 2.2. Generation of Traveling Wave

Multiple methods exist for generating traveling waves in stators. These include employing a ring-structured stator to mitigate reflections at the boundary and facilitate wave propagation utilizing a Langevin transducer to induce vibrations at one end of the stator while dampening them at the opposite end. This paper uses piezoelectric ceramic to generate traveling waves at the end of the stator by torsional vibration.

The traveling wave component generated at the excitation end will be reflected at the non-excitation section of the stator, changing the traveling wave propagation mode and forming a standing wave in the stator. Designing and improving the motor structure is necessary to achieve a relatively pure traveling wave in the stator.

### 2.3. Vibration-Absorbing Structure Design

**Figure 2:**
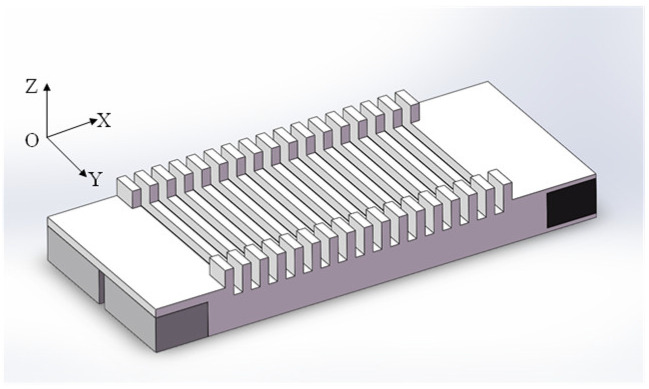
The first vibration-absorption structure.

A vibration-absorbing structure has been developed in light of the issues above with the motor’s structural design. This paper primarily utilizes passive damping-induced energy dissipation during plastic deformation to absorb reflected traveling waves. The key to reducing the standing wave ratio of the traveling wave motor and increasing its traveling wave component lies in fully utilizing the hysteretic characteristics of the damping material. This can be accomplished by enhancing the plastic deformation produced by the damping material, which efficiently absorbs reflected traveling waves via energy dissipation. Figure 2 shows that the unexcited end of the motor employs an identical structure to that of the excited piezoelectric ceramic. The stator’s vibration is transmitted through two upper and lower convex structures to the end damping material, which undergoes plastic deformation and effectively absorbs reflected traveling waves.

The structure depicted in Figure 2 can generate a certain degree of traveling wave components after conducting numerous finite element simulations. Its performance is unsatisfactory due to a standing wave ratio of 3.52, significantly exceeding the standard for traveling-wave linear motors. The standing wave component comprises 71.6%, while the traveling wave component comprises only 28.4%.

**Figure 3:**
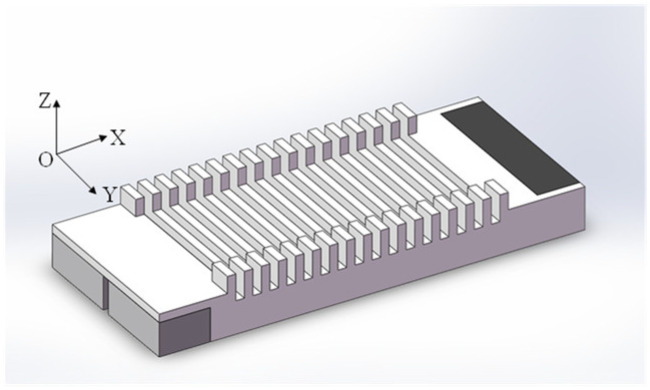
The second vibration-absorption structure.

Figure 3 shows the second vibration-absorption structure. A second vibration-absorbing structure is suggested to further reduce the standing wave ratio and component and produce a relatively pure traveling wave within the stator body. The arrangement conveys variations in vibrations to the damping material, resulting in opposing shear deformations along the Z-axis at both ends of the material.

Both structures use passive damping to absorb vibration at the end. In Figure 2 and Figure 3, all other parameters of the two motors are identical except for the vibration attenuation structure located at the end. Analyzing deformation in both configurations is necessary to determine the amplitude of the traveling wave component in the stator between the two structures and the efficacy of damping materials. The displacement data of each node in the damping material is extracted using ANSYS 2023 APDL platform. Due to the diverse structures of motors, direct comparison of damping material deformation is not feasible. The energy dissipation degree is reflected by comparing coefficient variation in damping material displacement.

The coefficient *CV* of variation for the deformation of the damping material is calculated as follows:

(1) $$CV=\frac{SD}{MN}\times 100\%$$

(2) $$SD=\sqrt{\frac{1}{N}\sum_{i=1}^{N}{\left(S_{i}-\mu \right)}^{2}}$$

(3) $$MN=\sqrt{\frac{\sum_{i=1}^{N}{S_{xi}}^{2}+\sum_{i=1}^{N}{S_{yi}}^{2}+\sum_{i=1}^{N}{S_{zi}}^{2}}{N^{2}}}$$

where *SD* represents the overall standard deviation of damping material deformation, and *MN* denotes the average total displacement value.

Table 1 shows the finite element simulation results. It is evident that the displacement of the damping material, which constitutes the second vibration-absorbing structure in three directions (x, y, and z), as well as the variation coefficient of combined displacement, is respectively, 2.04, 36.84, 2.29, and 2.21 times greater than those of motors belonging to the first structure. The standing wave ratio of the second structure is merely 41.5% of that in the first structure, indicating a significant reduction compared to its predecessor, while the proportion of the traveling wave component increased to 68.26%. Therefore, the traveling wave type finally adopts the second design scheme.

## 3. Operation Principle

### 3.1. Polarizations and Arrangements of PZTs

The conventional linear traveling-wave ultrasonic motor generates a traveling wave by utilizing the longitudinal vibration of the Langevin transducer to induce a bending deformation on the straight beam. This results in an elliptical motion trajectory for particles on the straight beam, which drives the mover to perform linear motion through frictional interaction with the stator. This paper presents a linear traveling-wave ultrasonic motor that generates torsional vibrations in lead zirconate titanate (PZT) piezoelectric ceramic by applying an alternating current with a 180° difference to two PZT ceramics embedded within a stator. The configuration activates the vibration of protruding structures on either side of the stator, facilitating the transmission of vibrations throughout the entire assembly. Further stimulation of the vertical protrusion at the non-excited end causes extensional deformation in the passive damping, while energy dissipation counteracts the traveling wave reflected from that end. The amplitude of the reflected traveling wave is greatly reduced, and the two columns traveling wave in the stator body are in opposite directions. The stator teeth move elliptically due to the superposition of traveling waves with different amplitudes, creating a composite wave with traveling and standing wave components.

**Figure 4:**
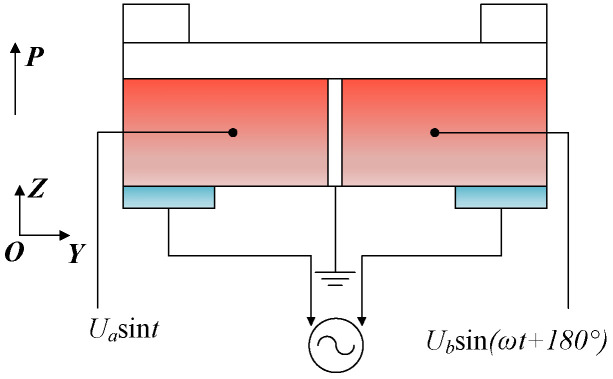
Excitation and detection scheme of PZT ceramics.

**Figure 5:**
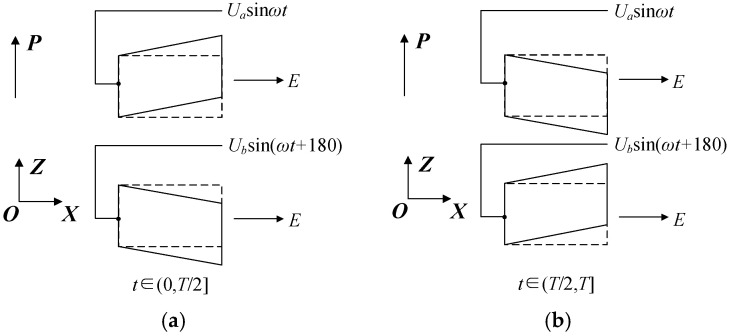
Deformation of excited PZT ceramics in different periods: (**a**) the deformation of PZT ceramics in the first half of the vibration cycle; (**b**) the deformation of PZT ceramics in the latter half of the vibration cycle.

The polarization direction of all piezoelectric ceramics is the positive direction of the Z-axis, as shown in Figure 4 and Figure 5. *U_a_* and *U_b_* are the amplitude of the voltage applied to the surface of the excited piezoelectric ceramics; *ω* is the angular frequency; T = 2*πf*, *f* is the frequency of power; *t* is the time; *P* is the polarization direction; and *E* is the electric field direction.

Figure 5a,b show excited PZTs’ deformation during one vibration cycle. The dotted line shows their initial shape, while the solid line shows their distorted shape under high-frequency alternating current excitation. Piezoelectric ceramics’ perpendicular polarization direction to the electric field generates d_15_ shear deformation, which excites the incident traveling wave.

### 3.2. Traveling Wave Equation of the Stator

In an unclosed structure, wave propagation is subject to reflection at the boundary of different media. Assuming that the incident wave excited by the piezoelectric ceramic can be expressed as follows, the wave equation in the motor can be derived:

(4) $$y_{1}=A_{1}\cos\left(\omega t-\frac{2\pi }{\lambda }x+\theta_{1}\right)$$

where *A*_1_—traveling wave amplitude generated by excitation of the PZT ceramics at the excitation end, *ω*—angular frequency, *t*—time, *λ*—the wavelength of stator wave, *x*—propagation distance of traveling wave, and *θ*_1_—the initial phase of the traveling wave generated by excitation of the PZT ceramics.

If the traveling wave experiences *θ*_2_ phase loss at the stator–damping material contact point due to unequal propagation, the reflected wave at the non-excitation end is as follows:

(5) $$y_{2}=A_{2}\cos\left(\omega t+\frac{2\pi }{\lambda }x+\theta_{2}\right)$$

where *A*_2_—the amplitude of the traveling wave reflected from the end of the stator and *A*_1_ > *A*_2_; then, the superimposed incident wave and reflected wave are as follows:

(6) $$\begin{array}{cc} y & =y_{1}+y_{2}\\ & =A_{1}\cos\left(\omega t-\frac{2\pi }{\lambda }x+\theta_{1}\right)+A_{2}\cos\left(\omega t+\frac{2\pi }{\lambda }x+\theta_{2}\right)\\ & =A_{2}\cos\left(\omega t-\frac{2\pi }{\lambda }x+\theta_{1}\right)+A_{2}\cos\left(\omega t+\frac{2\pi }{\lambda }x+\theta_{2}\right)+\left(A_{1}-A_{2}\right)\cos\left(\omega t-\frac{2\pi }{\lambda }x+\theta_{1}\right)\\ & =2A_{2}\cos(\frac{2\pi }{\lambda }x+\frac{\theta_{2}-\theta_{1}}{2})\cos(\omega t+\frac{\theta_{1}+\theta_{2}}{2})+\left(A_{1}-A_{2}\right)\cos\left(\omega t-\frac{2\pi }{\lambda }x+\theta_{1}\right) \end{array}$$

The synthetic wave equation formed in the stator has a standing wave component and a traveling wave component, where the standing wave component is $2A_{2}\cos[\omega t+(\theta_{1}+\theta_{2})/2]\cos[\omega t+(\theta_{1}+\theta_{2})/2]$ and the traveling wave component is $\left(A_{1}-A_{2}\right)\cos\left(\omega t-2\pi x/\lambda +\theta_{1}\right)$. Since *A*_1_ > *A*_2_, the final synthesized waveform in the stator is not a pure traveling wave but exists as a mixed wave comprising a traveling wave and standing wave.

At the same time, the traveling–standing wave in the stator can also be expressed as follows:

(7) $$\begin{array}{cc} y & =y_{1}+y_{2}\\ & =A_{1}\cos\left(\omega t-\frac{2\pi }{\lambda }x+\theta_{1}\right)+A_{2}\cos\left(\omega t+\frac{2\pi }{\lambda }x+\theta_{2}\right)\\ & =A(x)\cos[\omega t+\varphi (x)] \end{array}$$

where amplitude and initial phase of y are as follows:

(8) $$A(x)=\sqrt{A_{1}^{2}+A_{2}^{2}+2A_{1}A_{2}\cos(\frac{4\pi }{\lambda }x+\theta_{2}-\theta_{1})}$$

(9) $$\varphi (x)=\frac{\theta_{1}+\theta_{2}}{2}-\arctan\left[\frac{A_{1}-A_{2}}{A_{1}+A_{2}}\tan(\frac{2\pi }{\lambda }x+\frac{\theta_{2}-\theta_{1}}{2})\right]$$

Equation (8) shows that when $x=[2k+1+\left(\theta_{1}-\theta_{2}\right)/\pi ]\lambda /4$, the amplitude of *y* reaches the minimum value, which is *A*_1_ − *A*_2_. Meanwhile, when $x=[k+(\theta_{1}-\theta_{2})/2\pi ]\lambda /2$, the value reaches the maximum, which is *A*_1_ + *A*_2_. The standing wave ratio $\Gamma =(A_{1}+A_{2})/(A_{1}-A_{2})$ can be used to represent the amount of traveling waves. The closer the standing wave ratio *Γ* is to one, the higher the level of traveling wave presence.

### 3.3. Detection Scheme of the Traveling Wave

The traveling wave and standing wave components, the reflection coefficient of the traveling wave, and phase loss must be analyzed to facilitate the detection of the standing wave ratio in stators. Two thin PZT ceramics are attached to the bottom of the motor at wavelengths 5λ/8 and 7λ/8, respectively, and serve as detection ceramics. As the polarized PZT ceramic exhibits a linear relationship between amplitude and voltage within a specific resonance range, assuming that the measured voltages on the surface of PZT ceramics at wavelengths 5λ/8 and 7λ/8 are ${\dot{V}}_{1}=V_{1}∠\varphi_{1}$ and ${\dot{V}}_{2}=V_{2}∠\varphi_{2}$, respectively, then there exists the following:

(10) $$V_{1}^{2}={A_{1}}^{2}+{A_{2}}^{2}-2A_{1}A_{2}\sin(\theta_{2}-\theta_{1})$$

(11) $$V_{2}^{2}={A_{1}}^{2}+{A_{2}}^{2}+2A_{1}A_{2}\sin(\theta_{2}-\theta_{1})$$

(12) $$-\tan(\varphi_{1}-\frac{\theta_{1}+\theta_{2}}{2})=\frac{A_{1}-A_{2}}{A_{1}+A_{2}}\frac{1+\tan\frac{\theta_{2}-\theta_{1}}{2}}{1-\tan\frac{\theta_{2}-\theta_{1}}{2}}$$

(13) $$-\tan(\varphi_{2}-\frac{\theta_{1}+\theta_{2}}{2})=\frac{A_{1}-A_{2}}{A_{1}+A_{2}}\frac{-1+\tan\frac{\theta_{2}-\theta_{1}}{2}}{1+\tan\frac{\theta_{2}-\theta_{1}}{2}}$$

Combining (10)–(13), the numerical solution of *A*_1_, *A*_2_, *θ*_1_, and *θ*_2_ can be solved by the least square method so that the standing wave ratio *Γ* can be expressed as follows:

(14) $$\Gamma =\frac{1}{\sqrt{-\tan(\varphi_{1}-\frac{\theta_{1}+\theta_{2}}{2})\tan(\varphi_{2}-\frac{\theta_{1}+\theta_{2}}{2})}}$$

Similarly, (15)–(17) can be utilized to express the proportions of traveling wave *R_T_* and standing wave *R_S_*, as well as the reflection coefficient of traveling wave α:

(15) $$R_{T}=\frac{A_{1}-A_{2}}{2A_{2}+A_{1}-A_{2}}=\frac{A_{1}-A_{2}}{A_{1}+A_{2}}=\frac{1}{\Gamma }$$

(16) $$R_{s}=\frac{2A_{2}}{2A_{2}+A_{1}-A_{2}}=\frac{A_{1}+A_{2}-(A_{1}-A_{2})}{A_{1}+A_{2}}=\frac{\Gamma -1}{\Gamma }$$

(17) $$\alpha =\frac{A_{2}}{A_{1}}$$

Therefore, to expedite the motor design process and minimize experimental costs during actual implementation, two PZT ceramics with a spatial phase difference of λ/4 can be positioned on one side surface of the stator as a mechanism for detecting stator waveforms. It means that the PZT ceramics can be used to replace the laser Doppler vibrometer to a certain extent.

### 3.4. Formation of Ellipse Motion of Particles on Stator Surface

The linear ultrasonic motor utilizing a traveling wave employs the inverse piezoelectric effect of PZT ceramics to resonate the stator and generate a traveling wave in the stator body. This results in an elliptical trajectory of particles on the stator surface, which converts electrical energy into mechanical energy through contact between the stator teeth and the contact surface.

Based on the waveform equation of the stator, the stator transverse displacement can be determined as follows:

(18) $$u_{z}=A_{1}\cos\left(\omega t-kx+\theta_{1}\right)+A_{2}\cos\left(\omega t+kx+\theta_{2}\right)$$

where *u_z_*—lateral displacement of the stator and k=2π/λ—number of wave cycles per unit length in the direction of wave propagation. The longitudinal displacement of the stator can be expressed as follows:

(19) $$u_{x}=\frac{h}{2}\frac{\partial u_{z}}{\partial x}=\frac{hk}{2}A_{1}\sin\left(\omega t-kx+\theta_{1}\right)-\frac{hk}{2}A_{1}\sin\left(\omega t-kx+\theta_{1}\right)$$

where *u_x_*—longitudinal displacement of the stator, and *h*—distance from the stator’s neutral layer to the surface.

The order of ${\left(khu_{z}/2\right)}^{2}+{u_{x}}^{2}$ is as follows:

(20) $$\frac{h^{2}k^{2}}{4}{u_{z}}^{2}+{u_{x}}^{2}=\frac{h^{2}k^{2}}{4}({A_{1}}^{2}+{A_{2}}^{2})+\frac{h^{2}k^{2}}{2}A_{1}A_{2}\cos(2\omega t+\theta_{1}+\theta_{2})$$

The particle trajectory on the surface can be determined by solving Equation (20) in its entirety:

(21) $$\begin{array}{l} {\left(\frac{2u_{x}}{hk\sqrt{{A_{1}}^{2}+{A_{2}}^{2}+2A_{1}A_{2}\cos(2\omega t+\theta_{1}+\theta_{2})}}\right)}^{2}\\ +{\left(\frac{u_{z}}{\sqrt{{A_{1}}^{2}+{A_{2}}^{2}+2A_{1}A_{2}\cos(2\omega t+\theta_{1}+\theta_{2})}}\right)}^{2}=1 \end{array}$$

**Figure 6:**
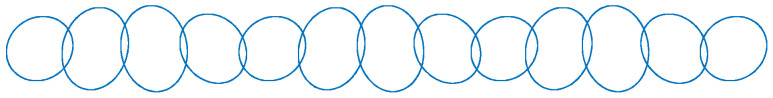
Ellipse trajectory distribution on the surface of the stator.

According to Equation (21), the particle’s motion trajectory in the *X*-*O*-*Z* plane is an ellipse. Furthermore, this equation can be utilized to depict the elliptical motion trajectories of surface particles at various positions on the stator, as illustrated in Figure 6:

As shown in Figure 6, each elliptical trajectory is spaced apart λ/8. Due to the variation in phase differences in both lateral and longitudinal displacements during wave propagation, it does not always remain at π/2, and the elliptical trajectory of the motor is not a standard one, as it varies between an oblique and a traditional ellipse. This results in a non-linear waveform envelope, with the standard ellipse being achieved only at maximum and minimum amplitudes. The surface amplitude of the stator undergoes periodic changes between the maximum value *A*_1_ *+ A*_2_ and minimum value *A*_1_ − *A*_2_. As the standing wave ratio decreases, the elliptical motion trajectory can drive loads more continuously and reduce impact with contact surfaces, making it superior to standing-wave linear motors in performance.

The expressions for the lateral and longitudinal velocities of surface particles can be further derived as follows:

(22) $$v_{z}=\frac{\partial u_{z}}{\partial t}=-A_{1}\omega \sin\left(\omega t-kx+\theta_{1}\right)-A_{2}\omega \sin\left(\omega t+kx+\theta_{2}\right)$$

(23) $$v_{x}=\frac{\partial u_{x}}{\partial t}=\frac{hk}{2}A_{1}\omega \cos\left(\omega t-kx+\theta_{1}\right)-\frac{hk}{2}A_{2}\omega \cos\left(\omega t-kx+\theta_{2}\right)$$

## 4. FEM Analysis

A three-dimensional model of the motor was established and meshed in ANSYS 2023 APDL platform finite element simulation software to analyze the motor’s operational state. Modal analysis, harmonic response analysis, and transient analysis are conducted to investigate the vibration characteristics of the motor.

### 4.1. Model Analysis

In this design, the stator is made of steel, the damping material is composed of silicon rubber, and the piezoelectric ceramic used is PZT-4. Table 2 shows the specific material parameters. By establishing a finite element model of the proposed motor stator in ANSYS software, the elastic body was modeled using SOLID45 elements, while the damping material was modeled using SOLID185 elements. To facilitate piezoelectric coupled-field analysis, the piezoelectric components were modeled using the 20-node high-order SOLID226 element. Compared with the older SOLID5 element, SOLID226 provides superior computational accuracy and solution precision.

The main structural parameters of the proposed motor are presented in Table 3.

**Figure 7:**
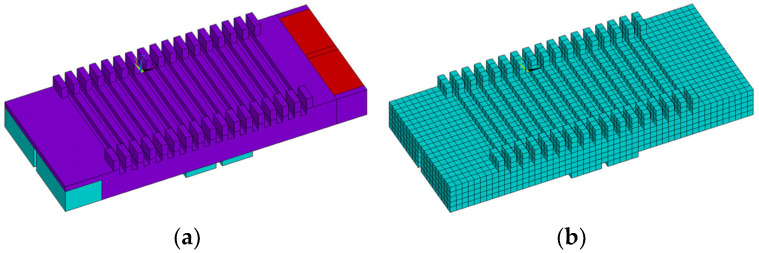
Stator modeling. (**a**) Finite element model. (**b**) Meshing model.

The parametric finite element model of the dual-traveling-wave linear ultrasonic motor stator based on the out-of-plane mode, together with its meshing result, are shown in Figure 7a and Figure 7b, respectively. A sweep meshing method was adopted to control mesh quality, resulting in a fully hexahedral mesh. The final model consisted of 7372 nodes and 5154 elements.

**Figure 8:**
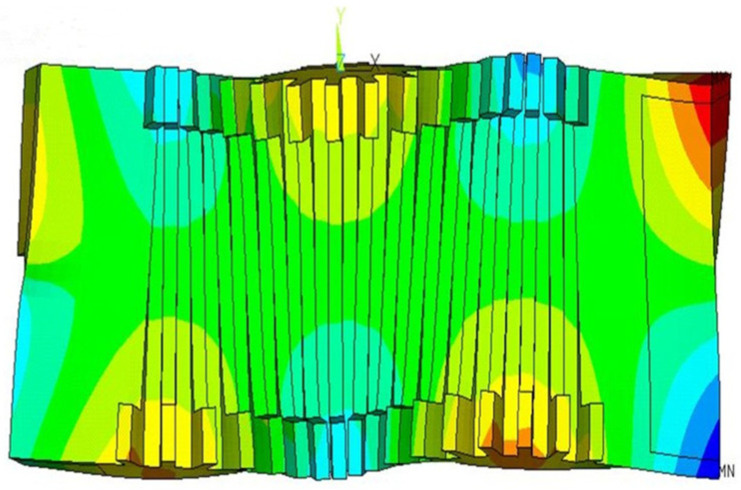
Vibration mode of the linear stator.

Based on this mesh, the modal characteristics of the stator in the ultrasonic frequency range were analyzed. The stator was maintained under free boundary conditions on all four sides, and the starting and ending frequencies for modal analysis were set to 20 kHz and 80 kHz, respectively. The Block Lanczos method was employed to extract the modal shapes. A total of 24 mode shapes was obtained within the frequency range of interest, among which the required thin-plate B(3,1) vibration mode was identified, as shown in Figure 8. The motor’s vibration mode B_31_ pertains to the stator, while its natural frequency oscillates at approximately 48 kHz. It can be seen that, in the central metallic elastic body of the stator, there are three nodal lines along the Y-axis and one nodal line along the X-axis. At the ends near the piezoelectric actuators, the displacements in the Z-axis direction exhibit completely opposite phases. The damping material at the tail of the stator shows the same characteristic. These features are consistent with the definition of an out-of-plane mode and are beneficial for deformation-based energy dissipation in the damping material.

### 4.2. Harmonic Response Analysis

The complete method is utilized to analyze the stator finite element model’s harmonic response and investigate its frequency response under continuous periodic voltage load. Based on the natural frequency of thin-plate *B*_31_ obtained through modal analysis, a frequency scanning range of interest is set between 48 kHz and 54 kHz. The loading sub-step is 120 with a loading interval of 50 Hz.

Two piezoelectric ceramics are subjected to a sinusoidal alternating current of equal amplitude but with a phase difference of 180°, and the resulting solution is obtained. The top node of the driving foot on one side of the stator is selected as the observation point for amplitude after completion.

**Figure 9:**
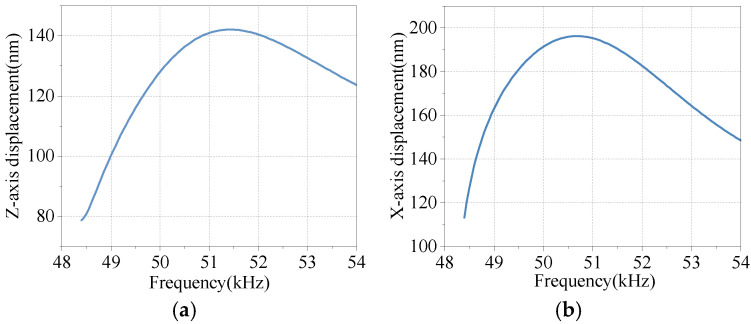
Frequency response of displacement: (**a**) Z-axis displacement response of measuring point; (**b**) X-axis displacement response of measuring point.

Figure 9a displays the displacement response of the observation point located at the driving foot in the Z-axis direction, while Figure 9b illustrates the displacement response of said observation point in the X-axis direction. Stator transverse and longitudinal displacements *u_z_* and *u_x_* reach their maximum values at 51.45 kHz excitation voltage, and damping conditions produce a rather flat harmonic response curve.

### 4.3. Transient Analysis

ANSYS conducts the transient dynamic analysis of the motor to investigate the movement process of particles on its surface.

**Figure 10:**
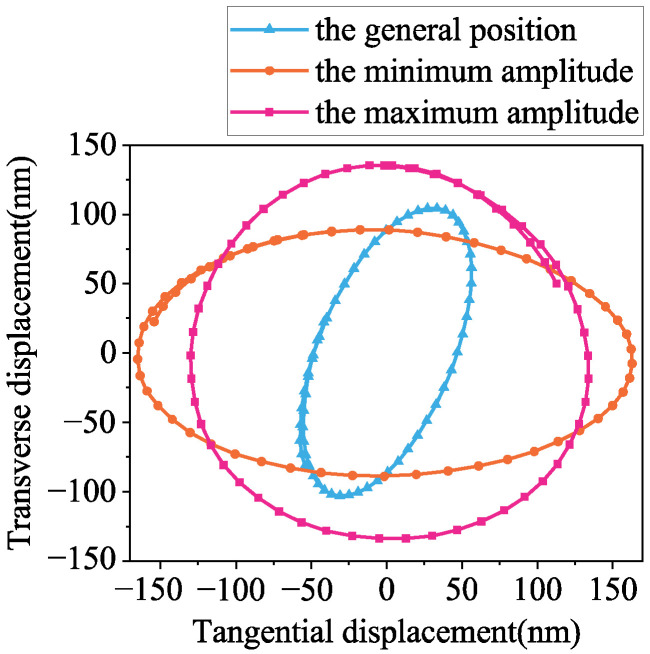
Motion trajectories of the teeth at different positions of the stator.

Figure 10 illustrates the extraction of X-axis and Z-axis amplitudes at maximum, minimum, and general positions of nodes to synthesize a closed displacement curve in the *X*-*Z* plane. Each point on the surface of the stator performs an elliptical motion trajectory.

### 4.4. Voltage–Speed Characteristics

**Figure 11:**
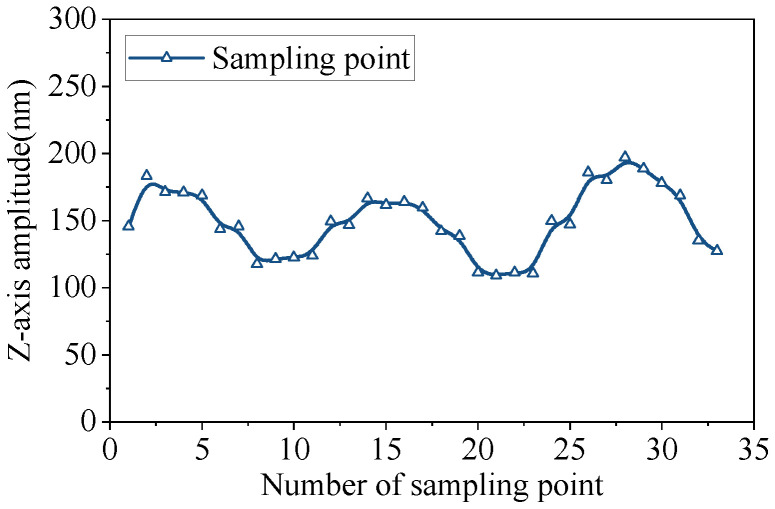
Amplitude distribution of driving feet.

Extracting the surface amplitude distribution of the stator to verify the generation of traveling waves in the stator is important. Figure 11 shows the distribution of the Z-axis amplitude of 33 nodes on the driving feet on one side of the stator.

In contrast to the standing-wave-type linear ultrasonic motor, the amplitude distribution of the linear traveling-wave ultrasonic motor does not intersect with the zero point. There are no standing wave nodes but rather relatively low and high points. This means that each point on the driving foot can provide a unidirectional driving force in space, ensuring the continuous motion of the mover.

Based on the amplitude data and the definition of the standing wave ratio, the standing wave ratio of the traveling wave motor reached approximately 1.73, with the traveling wave component accounting for roughly 57.8%.

### 4.5. Waveform Detection Voltage

**Figure 12:**
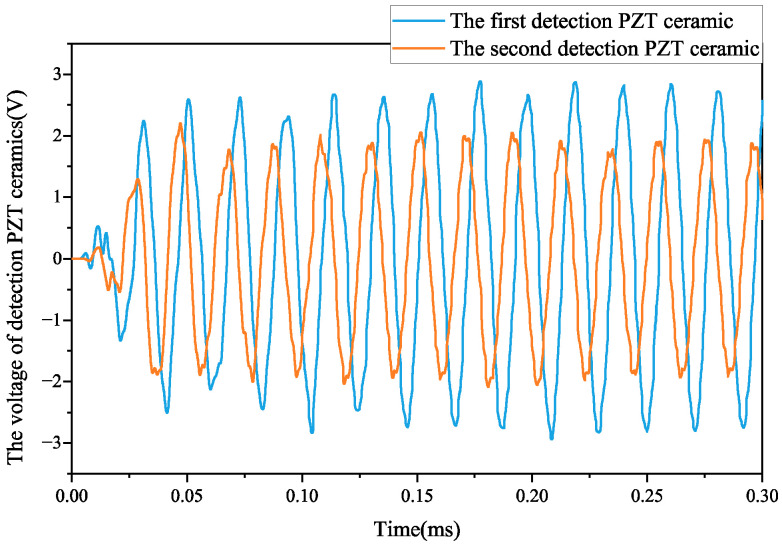
Waveform of voltage detected on PZT ceramics.

Figure 12 illustrates that the voltage waveforms detected on the two PZT ceramics at the bottom of the stator can be used to calculate parameters in the stator, including the standing wave ratio, traveling wave component, and traveling wave reflection coefficient.

**Figure 13:**
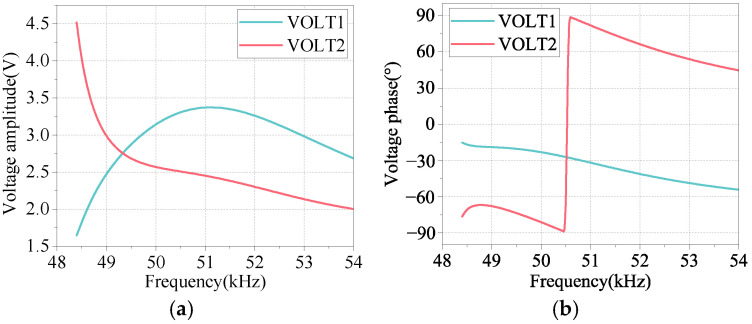
Voltage and phase of tested ceramics: (**a**) the magnitude of the detected voltage; (**b**) voltage and phase of tested ceramics.

Figure 13a displays the amplitude–frequency response curve of the detection voltage, while Figure 13b illustrates its phase–frequency response curve at the maximum amplitude point. The voltage amplitudes of the two test ceramics were measured to be 3.36 V and 2.39 V, respectively, with corresponding voltage phases of −36.15° and 74.66°, resulting in a phase difference of 110.81°, which closely approximates the expected pure traveling wave phase difference of 90°.

According to Equations (10)–(13), the calculated standing wave ratio is 1.662. The obtained value is close to the finite element analysis result, with a minimal error rate of 3.93%, demonstrating the feasibility and high measurement accuracy of the standing wave ratio detection scheme based on piezoelectric ceramics.

## 5. Prototype and Experiment

### 5.1. Fabrication of the Prototype

**Figure 14:**
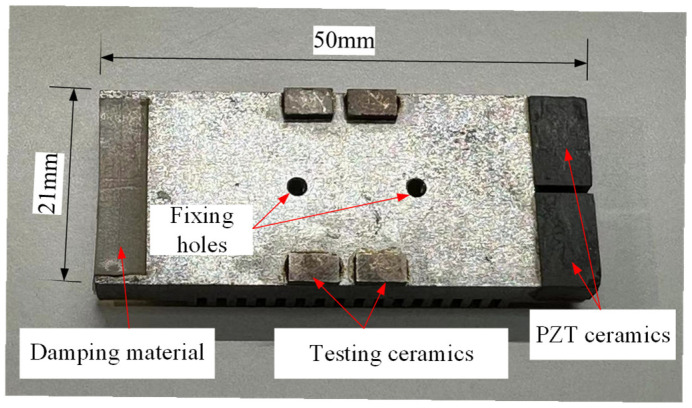
Stator of the motor.

The stator is processed using the finite element model and SolidWorks 2023 solid modeling. Figure 14 shows the completed prototype. The stator material is 45 steel, and silicone rubber is the damping material. An epoxy resin adhesive layer arranges various piezoelectric elements at predetermined positions. A pair of fixing holes is located in the middle of the stator, where the amplitude is the smallest, to facilitate the stator’s installation and fixing.

**Figure 15:**
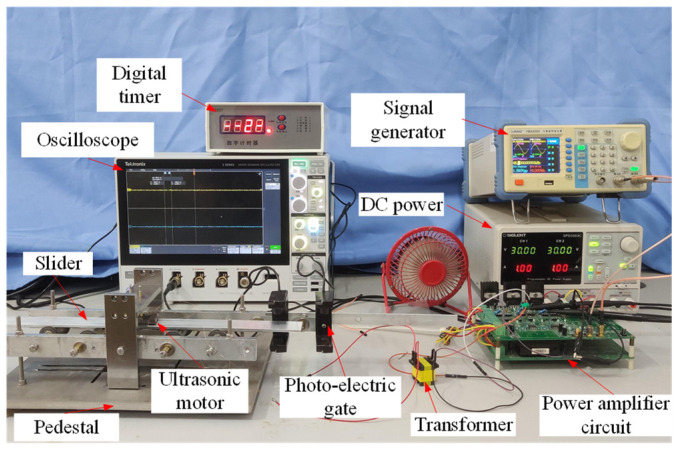
Experimental platform.

The experimental setup for the linear motor is depicted in Figure 15, comprising a direct current power supply (SIGLENT SPD3303C), signal generator (LVANG YB32020), power amplifier circuitry, transformer, oscilloscope (MDO34 3-BW-100), photoelectric gate, and digital timer.

**Figure 16:**
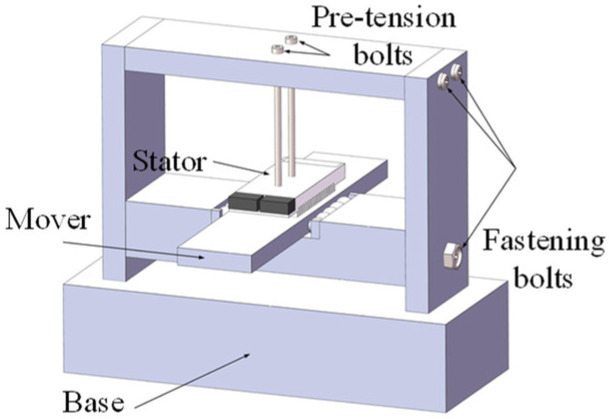
Motor drive platform.

Based on the prototype dimensions, a gate-type frame was designed for the experiment, as shown in Figure 16. A stainless-steel base with sufficient mass was used to ensure the operational stability of the motor. The frame was bolted to the base to support motor installation, and preloading holes were reserved at the top for stator fixation and preload application.

### 5.2. Impedance Characteristics of Ultrasonic Motor

**Figure 17:**
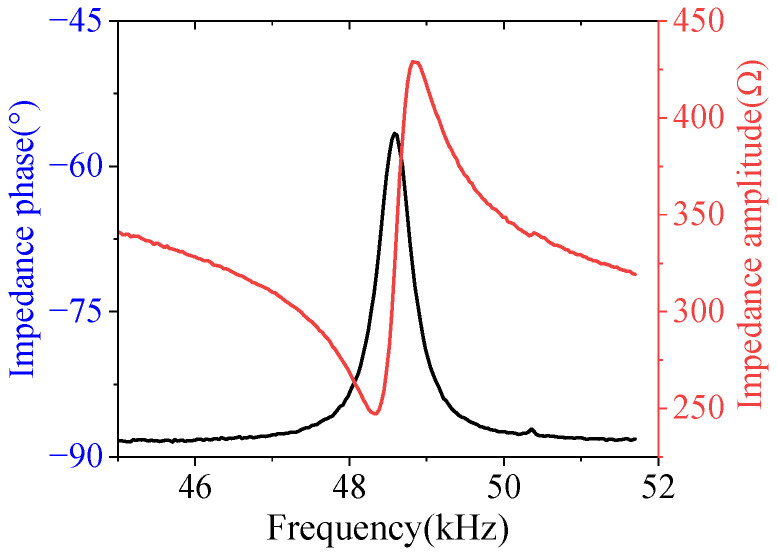
Impedance characteristic curve of ultrasonic motor.

The impedance analyzer tests the motor’s impedance characteristics, with a sweep frequency range selected from 45 kHz to 52 kHz based on modal analysis results. The voltage amplitudes applied on the two piezoelectric ceramics are equal. Figure 17 shows the motor’s impedance characteristics.

The maximum impedance amplitude of the ultrasonic motor is achieved at a frequency of 48.819 kHz, with a value of 428.9 Ω, while the maximum impedance phase occurs at 48.584 kHz. Due to the fixed hole position of the actual stator center and its connection to the platform, as well as errors in stator processing and adhesive layering, the resulting frequency sweep differs from the 49.298 kHz obtained through modal analysis.

### 5.3. The Relationship Between Frequency and Velocity

The ultrasonic motor operates based on the stator’s natural frequency and forced vibration. The amplitude response of the ultrasonic motor varies under different excitation frequencies, allowing for adjustment of its output characteristics by changing the input AC voltage frequency.

The slider utilized in this experiment is a polished aluminum alloy with dimensions of 35 cm in length, 3 cm in width, and 3 mm in thickness.

During the experiment, a voltage of 900 Vpp with a phase difference of 180° is applied to PZT ceramics, and the preload is 1 N. The frequency adjustment range is from 47.9 kHz to 48.7 kHz. Adjustments to the voltage frequency occur at intervals of 100 Hz. The final observation result is obtained by taking the average value from multiple measurements at each frequency point.

**Figure 18:**
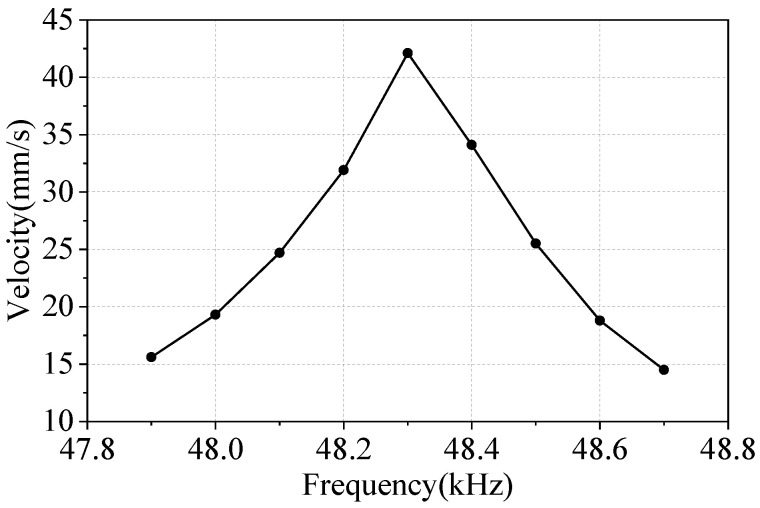
Frequency-Velocity characteristic curve.

Figure 18 shows the frequency–velocity characteristic curve of the motor. The linear output speed increases and subsequently decreases with an increase in frequency. At a voltage frequency of 48.3 kHz, the mover’s operating speed reaches its peak value at 42.1 mm/s.

### 5.4. The Relationship Between Voltage and Velocity

The inverse piezoelectric effect of piezoelectric elements exhibits linearity within a certain range. As the voltage increases, the vibration intensity of the piezoelectric element also increases, resulting in greater amplitude. The greater the friction that the stator driving teeth provide to the mover slider under the preload, the faster the linear motion speed of the mover. The desired motor speed can be obtained by increasing the voltage amplitude.

**Figure 19:**
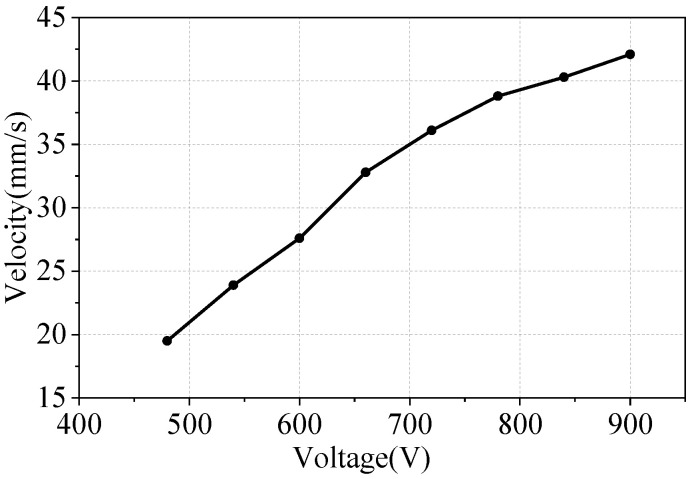
Voltage–velocity characteristics.

Figure 19 shows the voltage–velocity characteristics of the motor. The input voltage frequency remains at the resonant frequency of 48.3 kHz. Vpp is gradually adjusted from 480 V to 900 V under a preload of 1 N. The mover speed is measured multiple times for each voltage level to obtain an average value. The linear drive speed of the motor is proportional to the input voltage. As the voltage increases, the speed initially rises quickly, followed by a slower growth rate.

### 5.5. Linear Thrust Test

**Figure 20:**
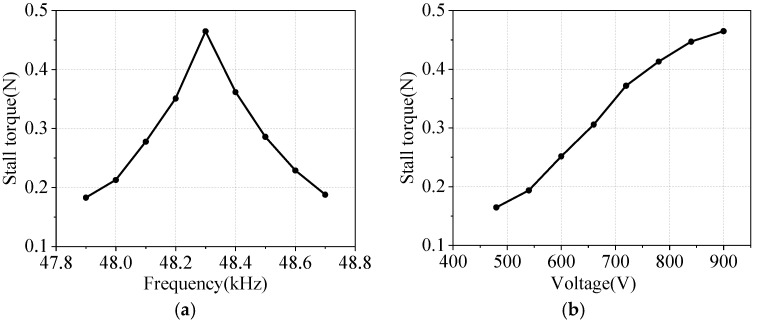
Frequency–stall torque or voltage–stall torque characteristic: (**a**) frequency–stall torque characteristic; (**b**) voltage–stall torque characteristic.

A digital torque meter measures the motor’s linear stall torque after its slider passes through the photoelectric door. Figure 20 shows the stall torque characteristics. When the voltage is maintained at 900 Vpp, the motor thrust reaches a maximum of 0.465 N at the resonant frequency. The linear thrust gradually increases as the voltage rises.

### 5.6. Mechanical Characteristics

**Figure 21:**
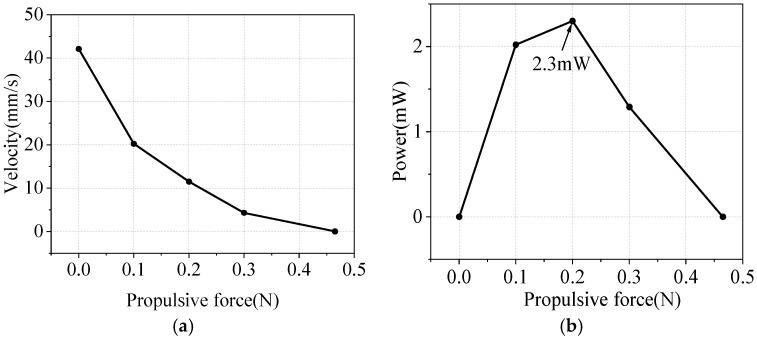
Mechanical characteristics: (**a**) thrust–linear speed; (**b**) thrust–output power.

Mechanical characterization of the out-of-plane mode traveling-wave ultrasonic motor is performed at a working frequency of 48.3 kHz and an excitation voltage of 900 Vpp to ascertain the motor’s actual load-bearing capacity. Weights with different mass specifications can be used to control the load in the experiment, and a fixed pulley is used to keep the force on the mover horizontal and avoid any negative effects of the load on contact. The characteristic curves of thrust linear speed and output power obtained from the test are presented in Figure 21a and Figure 21b, respectively. As the thrust increases, there is a downward trend in the mover’s running speed. When the mover’s speed decreases to zero due to obstruction by thrust tester, maximum thrust is achieved at 0.465 N. The motor’s output mechanical power initially increases and decreases with increasing thrust, peaking at 2.3 mW when the load is at 0.2 N.

### 5.7. Standing Wave Ratio Measurement Based on Testing Ceramics

The current method for detecting the standing wave ratio of the traveling-wave ultrasonic motor is primarily based on measuring surface amplitude distribution using a laser Doppler oscillator and then calculating it through amplitude data. However, this approach is cumbersome and requires high equipment precision, making real-time detection impossible. Two piezoelectric ceramics are mounted on the stator surface to facilitate detecting standing wave frequency. The proposed motor is easier to construct because the standing wave ratio, traveling wave component, and reflection coefficient can be calculated from the surface voltage.

**Figure 22:**
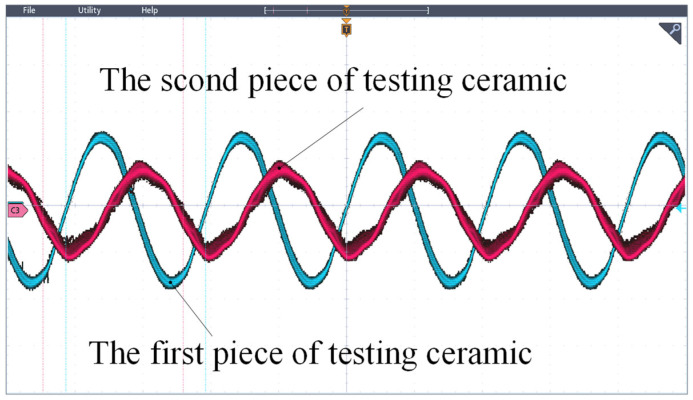
The oscilloscope detects the voltage waveform.

Figure 22 illustrates the waveform observed during motor operation. The two sinusoidal voltage sets have amplitudes of 3.92 V and 3.2 V, respectively, with a phase difference of approximately 60 degrees. The measured standing wave ratio for the motor is 1.81, slightly higher than that obtained from simulations by an error margin of about 4.62%.

Due to the deviation between the actual installation position of the test ceramic and its ideal position, a bonding layer exists between the stator and the test ceramic. Moreover, due to changes in boundary conditions caused by preloaded bolts, the accuracy of waveform test results is affected. The theoretical standing wave ratio differs from the measured value. An inspection ceramic installation groove should be incorporated at an appropriate location on the stator surface to mitigate experimental errors in stator manufacturing.

Table 4 shows comparisons between the proposed motor and previous works. The motor developed here is more suited for precision and high-torque applications since it is slightly smaller overall than the motor described in reference [27]. It also has a higher torque and a lower maximum speed. In contrast to the motor suggested in Reference [29], the motor in this paper is small overall and has a high speed. The proposed motor is easy to install, less expensive, and easy to achieve batch production, whereas the motors in reference [27] and reference [29] call for two brakes as a source of vibration.

## 6. Conclusions

In order to address the issues of complex structure and inconsistent two-phase excitation frequency in linear traveling-wave ultrasonic motors, a structural design that combines the out-of-plane vibration mode of thin-plate B(3,1) with damping material has been effectively implemented. This design reduces the reflection phenomenon of traveling waves in non-closed environments, achieves a standing wave ratio of 1.73, and has a higher traveling wave component.

The principal prototype has been developed, and the experimental design has been formulated. The stator impedance characteristics, frequency modulation and speed regulation characteristics, and the detection of voltage waveform during operation have all been examined through related experiments. The maximum linear pressing force is 0.465 N, and the maximum linear running speed is 42.1 mm/s.

## Figures and Tables

**Table 1:** Comparison of two vibration-absorbing structures.

	The First Structure	The Second Structure
CV of x-axis displacement	10.71%	21.84%
CV of y-axis displacement	12.61%	464.6%
CV of z-axis displacement	50.78%	116.35%
CV of total displacement	26.56%	58.76%
Standing wave ratio	3.52	1.46

**Table 2:** Motor modeling material parameters.

	PZT Ceramic	Steel Stator	Silicon Rubber Damping Material
Young’s modulus (GPa)	80	101	4.2 × 10^−3^
Density (kg/m^3^)	7600	8624	1510
Poisson’s ratio	0.33	0.373	0.45
Damping coefficient	-	-	0.05

**Table 3:** Structural parameters of the proposed motor.

Component	Length (mm)	Width (mm)	Thickness (mm)
Stator	50	21	5
PZT ceramic	10	6	4
Damping material	19	5	6

**Table 4:** Comparisons between the proposed motor and previous works.

Parameters		Proposed Motor	Tominaga et al. [28]	Yang et al. [29]
Maximum velocity (mm/s)		42.1	46.8	26
Maximum output thrust (N)		0.465	0.17	1.31
Stator size (mm)	Length	45	48	86
	Width	6	6	18
	Height	21	9	17.5
SWR		1.73	1.7	-

## Data Availability

The original contributions presented in this study are included in this article. Further inquiries can be directed to the corresponding author.
